# Detailed analysis of inbreeding in Tibetan sheep populations based on genome re-sequencing

**DOI:** 10.5713/ab.250600

**Published:** 2026-04-02

**Authors:** Lixia Sun, Chao Yuan, Tingting Guo, Bowen Chen, Zengkui Lu, Jianbin Liu

**Affiliations:** 1 Key Laboratory of Animal Genetics and Breeding on the Tibetan Plateau, Ministry of Agriculture and Rural Affairs, Lanzhou Institute of Husbandry and Pharmaceutical Sciences, Chinese Academy of Agricultural Sciences, Lanzhou, China; 2 Sheep Breeding Engineering Technology Research Center of Chinese Academy of Agricultural Sciences, Lanzhou, China

**Keywords:** Diversity Conservation, Inbreeding Calculation, Runs of Homozygosity, Tibetan Sheep

## Abstract

**Objective:**

This study investigates runs of homozygosity (ROH) in 11 Tibetan sheep using resequencing technology. The goal is to assess the inbreeding status and the timing of inbreeding events in Tibetan sheep. The study aims to deepen our understanding of the genomic diversity of Tibetan sheep by integrating analyses of gene flow and genetic diversity. The goal is to provide a theoretical foundation for conserving of genetic diversity and the optimizing genetic breeding strategies for Tibetan sheep.

**Methods:**

Sequencing data were obtained from 220 Tibetan sheep representing 11 different breeds across the provinces of Gansu, Qinghai, and the Tibet Autonomous Region. The size and distribution of genomic ROH fragments in these 11 Tibetan sheep populations were analyzed.

**Results:**

In 11 Tibetan sheep populations, 85% of the ROH were ranged from 0.5 Mb to 1 Mb in length. Only five populations (Kecai sheep, Ganjia sheep [GJ], Qiaoke sheep [QK], Tianjun white sheep [WT], and Tibetan Gangba black sheep) exhibited ROH segments longer than 3 Mb, although these instances were fairly rare. Chromosome length affects the number and size of ROH in the genome. Tibetan sheep have longer chromosomes 1, 2, and 3, leading to a higher occurrence of ROH. The F_ROH_ values were high for Tao sheep, GJ, and QK populations, while the lowest for Zhashijia sheep. Tibetan sheep populations harbor individuals with high inbreeding (F_ROH_>0.2), but overall inbreeding levels are low, primarily from earlier generations. The expected heterozygosity in the Tibetan sheep genome exceeds the observed heterozygosity, indirectly confirming the occurrence of inbreeding in Tibetan sheep populations. Gene flow is evident in various Tibetan sheep populations, particularly between Huoerba sheep and WT.

**Conclusion:**

The current breeding strategy for Tibetan sheep has room for improvement; therefore, breeding management and genetic diversity conservation should be prioritized in future programs.

## INTRODUCTION

The Tibetan sheep has a long history of development and is primarily found in the plateau regions adjacent to the mountains of the Qinghai-Tibet Plateau. According to statistics, there are 30 million Tibetan sheep on the 2.5 million km^2^ Tibetan plateau, with the Tibetan Autonomous Region and Qinghai provinces serving as the core production areas. Tibetan sheep are a vital source of production and livelihood and production for people living in pastoral regions. They provide abundant meat and wool, leather, and other products for the Tibetan population [1]. Moreover, they play a crucial role in the economic development and ecological protection of these production areas [2]. Tibetan sheep migrate and integrate with human activities, gradually spreading to 38 high-altitude regions across six provinces, including Tibet, Qinghai, Gansu, Yunnan, and Sichuan, on the Qinghai-Tibet Plateau [3,4]. Due to variations in geographical environments, socio-economic and ecological conditions, and breeding selection criteria, etc., Tibetan sheep exhibit significant differences in body size, appearance, production performance, and adaptability [5]. These complex conditions and factors have gradually led to the development of distinct ecological types of Tibetan sheep, resulting from prolonged geographical isolation. Given the highly plateau-adapted, semi-pastoral breed resources of Tibetan sheep, it is essential to prioritize biodiversity conservation, as well as the exploration and utilization of potential rare genetic loci [6,7]. Before developing a conservation plan for the population diversity of Tibetan sheep, it is essential to scientifically and accurately assess key parameters of population diversity, including inbreeding coefficients, genetic relationships, and levels of genomic heterozygosity. This assessment is vital for understanding the current status of the population’s genome.

The traditional method of calculating inbreeding coefficients based on pedigrees often results in discrepancies between observed and theoretical values, primarily due to incomplete pedigree records and calculation errors. In fact, even in well-established dairy cattle breeding programs worldwide, the error rate in pedigree records can be as high as 11%, significantly impacting critical aspects of livestock breeding [8]. With the development and widespread adoption of whole-genome sequencing technology, research on the population structure and breed differentiation of Tibetan sheep across various regions of China is progressively advancing at the genomic level. This technology is extensively used in numerous studies related to ancestral gene introgression, genetic diversity assessment, population historical dynamics, gene flow, and other aspects in animals, including pigs [9], cattle [10], and sheep [11]. The integration of whole-genome sequencing technology with bioinformatics analysis provides a more scientifically accurate genomic perspective for evaluating population diversity and inbreeding levels, as well as for the ecological conservation and genetic improvement of livestock and poultry populations [12].

The runs of homozygosity (ROH) segment in the genome is a homozygous regions inherited from the Homologous chromosome allele sequences of both parents [13]. Longer ROH segments on a chromosome originate from more recent common ancestors, while shorter segments derived from more distant common ancestors. During inbreeding, as the number of generations increases, ROH segments inherited from common ancestors become increasingly fragmented and rearranged in the offspring [14]. The length of ROH can be used to evaluate the degree of relatedness between individuals and assess genomic diversity, thereby facilitating the analysis of population structure and history [15,16]. Genomic ROH is also widely used for screening candidate genes for economic traits in livestock [17,18]. Since the concept of ROH was introduced, numerous researchers have employed this metric to evaluate the level of inbreeding in species such as cattle, sheep, and pigs, as well as analyze population history and structure [19]. In our previous research, we found evidence of common inbreeding among Tibetan sheep populations [20]. In order to better explore the detailed process of inbreeding events in these populations. In this study analyzes, the current status of inbreeding and genomic diversity in Tibetan sheep. On one hand, this aims to assess the genetic basis of populations adaptability; on the other hand, the scientific and accurate results can provide a theoretical foundation for the protection of genetic diversity and the genetic breeding management of high-altitude sheep breeds.

## MATERIALS AND METHODS

### Sample collection, DNA extraction and sequencing

**Figure 1:**
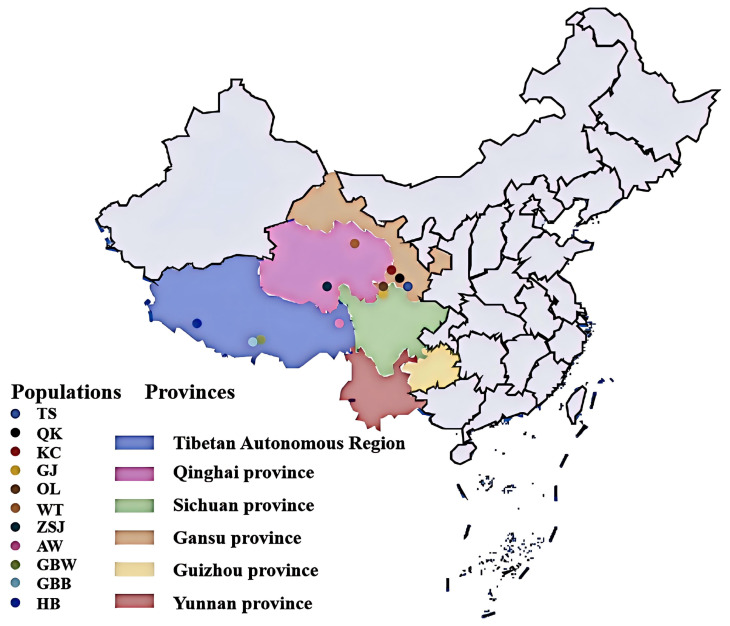
Sampling location information of Tibetan sheep. The gray areas on the map represent unsampled regions, while the other colors indicate sampled regions. The sampling locations of the 11 Tibetan sheep can be referenced by the distribution of the colored dots on the map. TS, Tao sheep; QK, Qiaoke sheep; KC, Kecai sheep; GJ, Ganjia sheep; OL, Oula sheep; WT, Tianjun white sheep; ZSJ, Zhashijia sheep; AW, Awang sheep; GBW, Gangba white sheep; GBB, Gangba black sheep; HB, Huoerba sheep.

A total of 220 blood samples were collected from 11 Tibetan sheep breeds across Gansu, Qinghai, and the Tibetan Autonomous Region [21]. Detailed information about the blood sample collection is provided in Supplement 1 and Figure 1.

Blood samples were collected from 11 Tibetan sheep populations, with more than two herds selected from each population. From these herds, 20 blood samples were randomly collected. Five milliliters of blood were drawn from the jugular vein into EDTA tubes and stored at −20°C. Genomic DNA was extracted using a DNA purification kit (K0512; Thermo Fisher Scientific). An appropriate volume of electrophoretic buffer (1X TAE or 0.5X TBE) was prepared to fill the electrophoresis chamber and to prepare the agarose gel. Five microliters of SYBR Gold dye were added to the gel, and five microliters of each DNA sample were loaded into the wells after the gel had solidified. DNA integrity was assessed based on the electrophoretic mobility and molecular sieving effect of DNA migrating through the agarose gel. Random genomic DNA fragments were enzymatically digested into short strands, which were then ligated to sequencing adapters. DNA fragments between 300 bp and 400 bp were selected for PCR amplification to construct the sequencing library. After library quality assessment, sequencing was performed on the HiSeq X10 platform, generating sequencing data with approximately 5X coverage depth.

### Data quality control and genome alignment

The original image data obtained through sequencing are converted into sequence data via base calling. The results are stored in FASTQ file format. To ensure data quality, the original data must be assessed before analysis, and noise should be minimized through filtering. The filtering parameters are set as follows: remove adapters and retain the remaining reads, remove reads containing Ns, and remove low-quality reads (where more than 50% of bases have a quality score of Q≤20).

Note: Adapter: Reads containing adapter sequences. N-containing: Reads with an N base ratio exceeding 10% in single-end reads. Low quality: Reads in which 50% or more of the base weight has a quality score (Q) of ≤20 in single-end reads. HQ clean: High-quality reads, defined as the remaining reads after filtering according to the above criteria.

We used BWA software to obtain high-quality reads, which were then aligned to the reference genome assembled by our team [22]. Sequencing was performed using Picard software, and duplicate short sequences were removed (http://sourceforge.net/projects/Picard/). To reduce the high mismatch error rate caused by insertions and deletions, GATK software was employed to realign the regions surrounding the variants, thereby obtaining accurate variant information [23]. Additionally, GATK was used to recalibrate base quality scores to ensure high-quality and reliable mutation data. Genome coverage and sequencing depth were analyzed using Bedtools (ver. 2.27.1) [24].

### Mutation detection and annotation

The processed alignment files were subjected to variant detection across multiple samples using the UnifiedGenotyper module in GATK software (ver. 3.4-46). Detected variants were filtered using the VariantFiltration tool with the following parameters: --Window size 4, -filter “QD<4.0 || FS>60.0 || MQ<40.0 “, -G_filter “GQ<20” (QD: Variant Confidence/Quality by Depth; FS: Phred-scaled p-value using Fisher’s exact test to detect strand bias; MQ: RMS Mapping Quality; GQ: Genotype Quality). Finally, ANNOVAR was utilized for the functional annotation of the identified variants [25].

### Runs of homozygosity detection and analysis

We used VCFtools version 0.1.14 to filter InDels and PLINK software to identify specific SNPs [26,27]. The analysis employed a sliding window approach along the chromosome to detect ROHs. The PLINK parameters were set as follows: --homozyg-kb 500; --homozyg-snp 20; --homozyg-window-snp 50; --homozyg-window-threshold 0.05; --homozyg-window-het 1; --homozyg-density 50; and --homozyg-gap 100. The detected ROHs were classified into three categories based on their length: Class A (short ROHs) for lengths less than 1 Mb, Class B (medium ROHs) for lengths between 1 and 3 Mb, and Class C (long ROHs) for lengths greater than 3 Mb.

### Runs of homozygosity inbreeding coefficient analysis

Inbreeding frequently occurs within populations, leading to an increased proportion of homozygotes and a decreased proportion of heterozygotes. To further investigate the history and extent of inbreeding in the genome of Tibetan sheep, we calculated the genomic inbreeding coefficient (F_ROH_) using PLINK software, based on the results of sample ROH detection. The formula for calculating F_ROH_ is as follows:


(1) $$F_{ROH}=\frac{\sum L_{ROH}}{L_{AUTO}}$$

L_ROH_ is the length of ROH in the genome; L_AUTO_ is the total length of autosomes covered by SNPs.

### Time analysis of inbreeding

We infer the level of inbreeding based on the length of the ROH fragments, using the formula: L_ROH = 100/(2 g), where l represents the centimorgan (cM) length of the ROH. This calculation assumes that all recombination rates at the same distance on the genome are constant (1 Mb = 1 cM), and g = the number of generations.

### Gene flow calculation

Gene flow analysis was conducted using the D-test. D-statistics, also known as the ABBA-BABA test, is a method used to detect significant non-conforming tree events caused by admixture. Treat the argali as an outgroup in the analysis, use PAUP software to construct a phylogenetic tree that includes outgroups, and use the topology of this evolutionary tree. Then, apply the Dtrios module of Dsuite to perform F4-ratio testing and the F-branch module of Dsuite for F-branch analysis, with a significance threshold of 0.05 for p-values. Finally, based on the Z-score and p-value provided by the results, select results with |Z|≥3 and p<0.05, and visualize the results using the built-in Dsuite scripts “plot_d.rb” and “plot_f4ratio.rb.” [28].

### Calculation of observed heterozygosity, expected heterozygosity and F_IS values

For the filtered SNP loci, the PLINK software was used to calculate the observed heterozygosity (Ho) and expected heterozygosity (He) of the populations [27]. The degree of deviation of He and Ho (F_IS = 1–Ho/He) can effectively evaluate the degree of inbreeding.

## RESULTS

### Sequencing data and genome-wide genetic variation

In this study, a total of 10,884,454 SNPs were identified from 220 Tibetan sheep individuals, representing 11 distinct Tibetan sheep breeds, with 20 individuals sampled from each breed (Table 1). The breeds from Gansu Province include Tao sheep (TS), Kecai sheep (KC), Ganjia sheep (GJ), Qiaoke sheep (QK), and Oula sheep (OL). From Qinghai Province, the breeds sampled were Zhashijia sheep (ZSJ), Tianjun white sheep (WT), Tibetan Gangba black sheep (GBB), Gangba white sheep (GBW), Huoerba sheep (HB), and Awang sheep (AW). The number of SNPs detected in the 11 whole-genome sequencing samples and their respective sampling locations are presented in Supplement 1 Genome variation annotation results are shown in Supplement 2, indicating that the majority of variations occur in intergenic regions, accounting for 60% to 72% of total genome variation. Intronic regions represent the second most significant category, comprising 30% to 36% of the variations. Additionally, minimal variation was observed in certain overlapping regions, with only 0.0003% to 0.0007% of variations occurring within 2 base pairs of exonic regions and variable splice sites. Overlap in the 5’ untranslated region (UTR) and 3’ UTR ranged from 0.003% to 0.004%.

### Genomic runs of homozygosity distribution and F_ROH_ calculation

**Figure 2:**
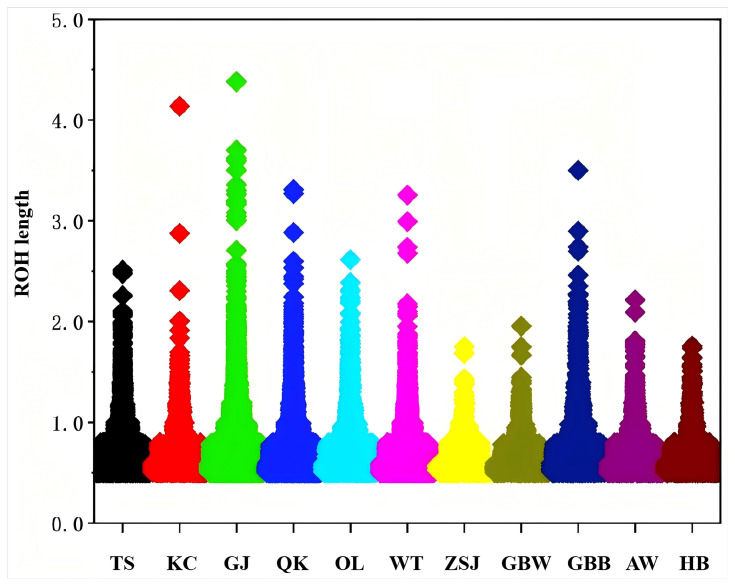
Genomic ROH length distribution in Tibetan sheep populations. ROH, runs of homozygosity; TS, Tao sheep; KC, Kecai sheep; GJ, Ganjia sheep; QK, Qiaoke sheep; OL, Oula sheep; WT, Tianjun white sheep; ZSJ, Zhashijia sheep; GBW, Gangba white sheep; GBB, Gangba black sheep; AW, Awang sheep; HB, Huoerba sheep.

The size and distribution of ROH fragments in the genomes of 11 Tibetan sheep populations ranged from 0.5 to 1 Mb. The detected ROHs were classified into three distinct length categories: class A (0.5–1 Mb), class B (1–3 Mb), and class C (>3 Mb). The results indicated that the average length of ROHs among the 11 Tibetan sheep populations ranged from 0.6 to 0.8 megabases (Mb). Additionally, the ROHs in these populations were primarily of type A, ranging from 0.5 to 1 Mb, accounting for more than 85%. This suggests that most inbreeding occurred in distant generations (Table 2). Only the KC, GJ, QK, WT, and GBB populations exhibited ROH fragments longer than 3 Mb (type C), although their proportions were relatively small. Significant differences were observed in the number of ROH fragments detected among the various populations. GJ, QK, and WT had the highest counts of ROH fragments, while ZSJ and HB had the lowest. The proportions of ROH types A, B, and C in GJ and GBB were similar; however, the number of ROH fragments of all three types in GBB was significantly higher than in GJ. Inbreeding events within the GBB population primarily occurred in recent generations. The mean number of ROHs detected was highest in the GJ population and lowest in the ZSJ population. The standard deviation (SD) of the number of ROHs detected across the 11 populations was substantial, indicating significant variability in the number of individual ROHs within the species (Table 2, Figure 2). The average ROH length was longest in the GJ population and shortest in the ZSJ population. The larger SD and coefficient of variation (CV) values for genome-wide ROH length among individuals within populations suggest greater dispersion of ROH length and lower uniformity in the level of inbreeding (Table 3).

**Figure 3:**
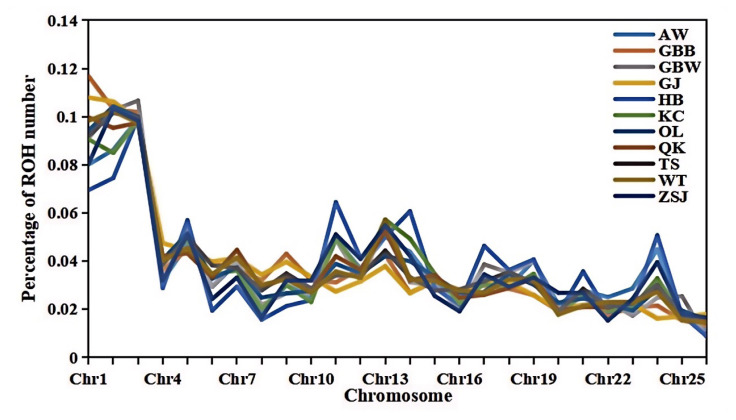
Distribution of ROH in chromosomes of Tibetan sheep populations. ROH, runs of homozygosity; AW, Awang sheep; GBB, Gangba black sheep; GBW, Gangba white sheep; GJ, Ganjia sheep; HB, Huoerba sheep; KC, Kecai sheep; OL, Oula sheep; QK, Qiaoke sheep; TS, Tao sheep; WT, Tianjun white sheep; ZSJ, Zhashijia sheep.

Overall, the percentage of ROH varied across different chromosomes; however, the general trend remained consistent. The percentage of ROH was higher on chromosomes 1, 2, and 3, while it was relatively lower on chromosomes 8, 22, and 26 (Figure 3). The primary reason for the varying proportions of ROH across different chromosomes in Tibetan sheep is the disparity in chromosome lengths. Chromosomes 1, 2, and 3 are significantly longer than the others. Consequently, even if ROH generated by inbreeding is randomly distributed among chromosomes, the number of ROH present on each chromosome will differ.

**Figure 4:**
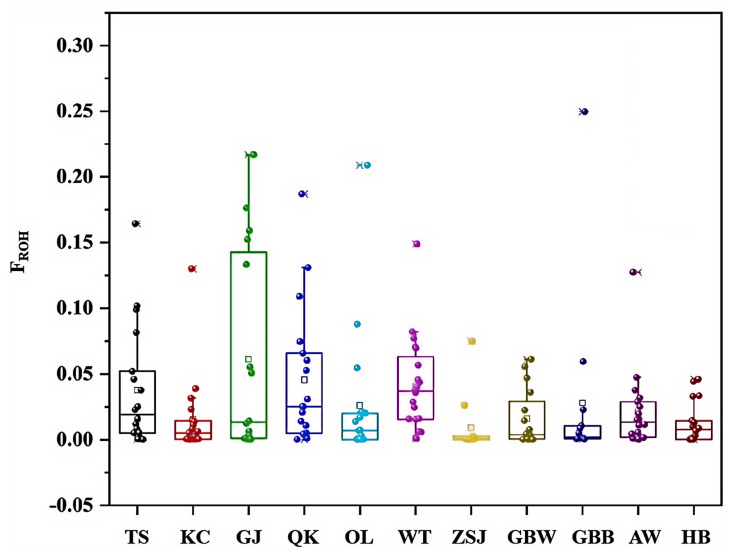
F_ROH_ values of Tibetan sheep populations. The lower and upper edges of the box indicate the first and third quartiles, respectively. The horizontal line inside the box represents the median. The lines extending from the top and bottom ends of the box represent the maximum and minimum values. ROH, runs of homozygosity; TS, Tao sheep; KC, Kecai sheep; GJ, Ganjia sheep; QK, Qiaoke sheep; OL, Oula sheep; WT, Tianjun white sheep; ZSJ, Zhashijia sheep; GBW, Gangba white sheep; GBB, Gangba black sheep; AW, Awang sheep; HB, Huoerba sheep.

The inbreeding coefficients of 11 Tibetan sheep populations were calculated using the ROH fragments detected in their genomes. The mean F_ROH_ coefficients for these populations ranged from 0.009 to 0.1. Among them, the TS, GJ, and QK populations exhibited the highest inbreeding coefficients, while the ZSJ population showed the lowest. Individuals with F_ROH_ values greater than 0.2 were identified in the GJ, OL, and GBB populations, indicating severe inbreeding in these individuals (Table 4). Additionally, Figure 4 illustrates that both Tibetan sheep populations display a wider range of F_ROH_ values, with the GJ and QK populations showing the greatest dispersion.

**Figure 5:**
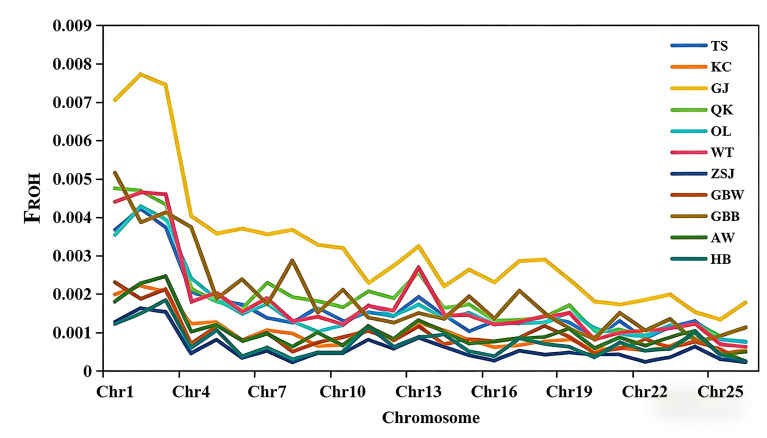
Distribution of F_ROH_ values on different chromosomes of Tibetan sheep populations. ROH, runs of homozygosity; TS, Tao sheep; KC, Kecai sheep; GJ, Ganjia sheep; QK, Qiaoke sheep; OL, Oula sheep; WT, Tianjun white sheep; ZSJ, Zhashijia sheep; GBW, Gangba white sheep; GBB, Gangba black sheep; AW, Awang sheep; HB, Huoerba sheep.

The F_ROH_ values of various chromosomes across 11 populations were analyzed. Although the F_ROH_ values varied among the Tibetan sheep populations and across different chromosomes, the overall trend remained consistent. The highest F_ROH_ values were observed on chromosomes 1, 2, and 3, while the lowest values were found on chromosomes 9, 23, and 26. Notably, the F_ROH_ values on chromosomes 9 and 15 exhibited differing trends among the populations (Figure 5).

**Figure 6:**
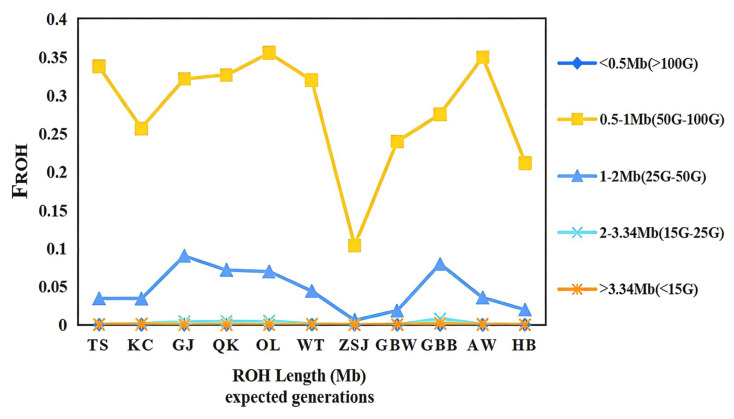
The generation and F_ROH_ values of inbreeding events in Tibetan sheep populations. ROH, runs of homozygosity; TS, Tao sheep; KC, Kecai sheep; GJ, Ganjia sheep; QK, Qiaoke sheep; OL, Oula sheep; WT, Tianjun white sheep; ZSJ, Zhashijia sheep; GBW, Gangba white sheep; GBB, Gangba black sheep; AW, Awang sheep; HB, Huoerba sheep.

To further analyze the duration of inbreeding, predictions were made based on the length of the ROH fragments. The analysis examined the relationship between different inbreeding durations and inbreeding coefficients. It was found that inbreeding among 11 Tibetan sheep populations occurred between the 50th and 100th generations. Notably, the ZSJ population exhibited the lowest frequency of inbreeding events, while the TS, OL, and AW populations demonstrated significantly higher frequencies compared to the others. Additionally, the GBB population experienced a markedly higher frequency of inbreeding events during the 15th to 25th generations (Figure 6).

### Gene flow detection

**Figure 7:**
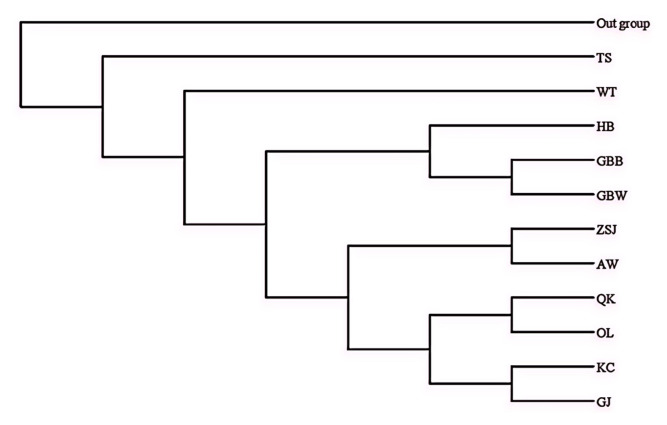
Phylogenetic tree of Tibetan sheep populations. The branches of a phylogenetic tree represent evolutionary clades, and species relationships can be inferred from branch lengths and branching points. Branch length indicates the amount of evolutionary change; shorter lengths indicate smaller differences and a closer evolutionary distance. TS, Tao sheep; WT, Tianjun white sheep; HB, Huoerba sheep; GBB, Gangba black sheep; GBW, Gangba white sheep; ZSJ, Zhashijia sheep; AW, Awang sheep; QK, Qiaoke sheep; OL, Oula sheep; KC, Kecai sheep; GJ, Ganjia sheep.

**Figure 8:**
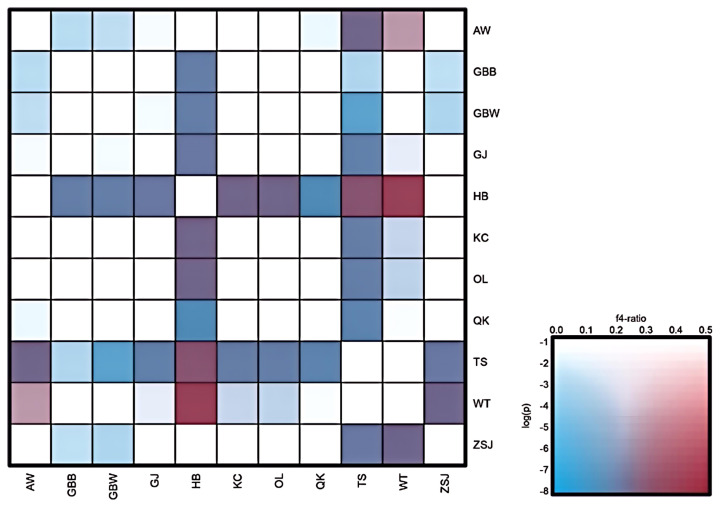
Gene flow determined using the F4-ratio. The F4-ratio statistic is an important metric used by Dsuite to infer the strength of gene flow between populations or species. A significantly positive value indicates the presence of gene flow among populations. Under the premise of a significant D value, a higher F4-ratio implies a larger proportion of genetic admixture. AW, Awang sheep; GBB, Gangba black sheep; GBW, Gangba white sheep; GJ, Ganjia sheep; HB, Huoerba sheep; KC, Kecai sheep; OL, Oula sheep; QK, Qiaoke sheep; TS, Tao sheep; WT, Tianjun white sheep; ZSJ, Zhashijia sheep.

**Figure 9:**
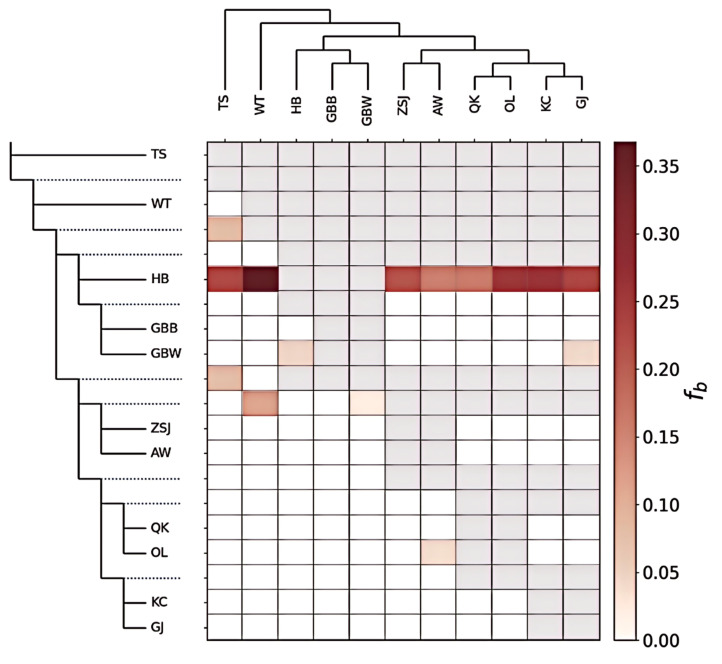
Gene flow determined using the f-branch method. The tree is displayed in an "expanded" form alongthe y axis, so that each branch, including internal branches, points to a corresponding row in the matrix with inferred f-branch statistics. The Fbranch program outputs a matrix with f-branch statistic values for each branch on the tree, including internal branches, reflecting excess allele sharing with each valid population or species P3. In the matrix, the shading intensity indicates the proportion of admixture: darker colors correspond to higher admixture proportions, while lighter colors indicate lower admixture proportions. TS, Tao sheep; WT, Tianjun whitesheep; HB, Huoerba sheep; GBB, Gangba black sheep; GBW, Gangba white sheep; ZSJ, Zhashijia sheep; AW, Awang sheep; QK, Qiaoke sheep; OL, Oula sheep; KC, Kecai sheep; GJ, Ganjia sheep.

According to the verification of the differentiation time in the phylogenetic tree, it can be observed that the differentiation time of the Argali sheep is earlier than that of the Tibetan sheep population, and it can be used as an outgroup for Tibetan sheep (Figure 7). By evaluating the gene flow patterns among the Tibetan sheep populations, we observe that the TS and HB populations exhibit more frequent gene flow with other populations. The WT and ZSJ groups also show limited gene flow with other populations, while the QK population displays the least gene exchange. Notably, there is clear gene exchange between the HB and WT groups (Figure 8). The f4-branch analysis indicates that HB shows no gene flow with the GBB, GBW populations, but shares gene flow with the remaining eight populations, with strong gene flow evident between HB and WT. The F4-branch analysis further corroborates the f4-ratio results (Figure 9).

### Diversity analysis

The changes in population genetic diversity influenced by inbreeding and gene flow were assessed by examining the He and Ho values in Tibetan sheep populations. The results showed that the Ho values for all Tibetan sheep populations ranged from 0.30 to 0.35, while the He values ranged from 0.15 to 0.25. Compared to the He value, the Ho value remains consistently low, indicating a lower level of genetic diversity within the population. The F_IS value can also effectively evaluate the homozygous status of the population genome. According to the analysis of population inbreeding degree, the range of F_IS values for these 11 populations is 0.314 to 0.418, which also suggests the accumulation of homozygotes within the population (Table 5).

## DISCUSSION

The occurrence of inbreeding events within a population leads to the formation of ROH in genomic chromosomes. The distribution, length, and other characteristics of these ROH segments reflect various structural features of the genome. Numerous studies have demonstrated that ROH segments can be used to assess the level of inbreeding in different livestock populations. The formation and selection of short ROH segments are associated with genomic linkage disequilibrium. Under the influence of selection, loci linked to economically important or phenotypic traits become fixed, along with adjacent genomic regions, resulting in the formation of contiguous homozygous segments within the genome [17]. In the sheep genome, strong linkage disequilibrium can extend up to 100 Kb. Therefore, it is essential to exclude short ROH segments in the sheep genome that arise due to this strong linkage disequilibrium. In this study, the minimum length for detecting ROH was established at 500 Kb [29]. The results of ROH detection in Tibetan sheep are consistent with previous studies, indicating that ROH segments typically range from 1 to 2 Mb in length, and the F_ROH_ of the population is relatively low [30]. The high proportion of short ROH reflects that ancient inbreeding event is not recent. Another reason for the abundant presence of short ROH segments in the Tibetan sheep genome may be that long ROH segments, generated by recent inbreeding, can be interrupted and recombined by various random factors during transmission, leading in the formation of short ROH segments [31]. The results of this study on the ROH of Tibetan sheep are consistent with the average F_ROH_ value of Asian sheep studied by Nosrati et al [32], which is around 0.1. The slight difference is that we found individuals with FROH higher than 0.2 in the Tibetan sheep population. This result also suggests that ranch managers need more scientific breeding management methods. The F_IS values are consistent with the ROH analyses, with all 11 sheep populations indicating inbreeding. The Ho across populations (0.173–0.199) is significantly lower than the He (0.290–0.306), indicating a buildup of homozygosity. Among them, the ZSJ population shows the highest level of inbreeding, suggesting that its genetic diversity loss is the most severe, which may be due to small population effects or stringent selection practices; by contrast, the QK population appears comparatively healthier, implying more effective gene flow or management practices. Notably, all populations have F_IS values exceeding 0.3, and together with the ROH analyses, the substantial accumulation of homozygotes is likely driven by ancient inbreeding. The results of this study indicate that the number and length of short ROH segments in the WT, GJ, and QK populations are greater than in other populations. This finding suggests that these three populations have experienced frequent inbreeding events and have been subjected to stronger selection pressures during the breeding process, resulting in a higher number of ROH formed by the fixation of trait-related loci. Furthermore, the study identified fewer long ROH segments, which were present only in the GBB, GJ, KC, QK, and WT populations, with GJ exhibiting the highest number of long ROH segments. Long ROH segments indicate a high degree of genomic homozygosity, primarily generated by successive inbreeding, and are less influenced by linkage disequilibrium and random effects [33]. The lengths and frequencies of genomic segments in the GBB and GBW populations are quite similar, likely due to their close geographical proximity within the Tibetan region, as well as comparable altitudes, ecological environments, and feeding conditions. Furthermore, gene flow occurs among TS population and HB populations and other populations, with partial gene exchange also observed for the WT, AW, QK, and OL groups. These patterns are largely related to the migration of Tibetan sheep populations. Tibetan sheep are thought to have accompanied prehistoric humans as they carried them onto the Qinghai-Tibet Plateau, forming the Qinghai subpopulation of Tibetan sheep. Subsequently, prehistoric humans, via the Silk Road corridor, continued to migrate and settle in the interior of the Qinghai-Tibet Plateau, leading to the dispersal of the Qinghai subpopulation into Tibet and the formation of the Tibetan subpopulation. Throughout this process, Tibetan sheep populations experienced varying degrees of gene flow between them [3]. Numerous studies have demonstrated that interspecies gene flow is a significant driver of species evolution [34]. Previous studies conducted by our group on the population structure of the Tibetan sheep genome have confirmed that most populations exhibit moderate to low levels of genetic differentiation. This research highlights the evolutionary relationships between the geographic locations of sheep populations and their genomic genetic distances [35].

Natural evolution and human activities significantly influence species distribution and population dynamics. The pronounced impact of humans can disrupt interactions between populations and intra-species selection. Selective breeding that favors individuals with superior economic traits can increase selection intensity and lead to frequent inbreeding within populations, resulting in reduced genomic heterozygosity. Populations with higher genetic diversity often exhibit greater environmental adaptability, resilience to disturbances, and enhanced evolutionary potential. He refers to the probability that an individual in a population is heterozygous at a given locus, whereas Ho represents the proportion of individuals in the population that are heterozygous at that locus. When He>Ho, it suggests possible inbreeding within the population or the influence of artificial selection; when He<Ho, it may indicate the introduction of new genetic variation [36]. This study found that the He of Tibetan sheep genomes is generally higher than the Ho, which aligns with the findings of Shi et al [37]. In our previous study, there was a mixture of principal component analysis and population structure analysis of Tibetan sheep population, and there was gene exchange in the population. These results are also consistent with our study, there is some gene flow between populations [35]. The number of haplotype sites in the Tibetan sheep genome exceeds that of polymorphic sites, characterized by a higher frequency of alleles at moderate frequencies and a lower frequency of rare alleles. The population appears to be under balancing selection, exhibiting low genetic diversity. This phenomenon may be linked to population management practices, as Tibetan sheep are primarily raised through grazing. In a closed environment, some individuals experience significant inbreeding, leading to a loss of heterozygosity and the formation of ROH segments, which ultimately reduces the overall level of population diversity [38]. This finding is consistent with the results of the ROH analysis, which indicate that the Tibetan sheep population exhibits low to moderate levels of inbreeding, although severe inbreeding is observed in some individuals across all Tibetan sheep populations.

The conservation of genetic diversity within Tibetan sheep populations is an urgent issue that demands immediate attention. Among the 11 Tibetan sheep populations studied, varying degrees of genetic diversity decline were observed, with the TS and GJ populations exhibiting higher levels of inbreeding and the lowest diversity. A commonly employed strategy for preserving diversity is the establishment of live breeding farms. For populations with relatively high genetic diversity, the creation of conservation farms can directly enhance and maintain their genetic variation. For populations with low genetic diversity, such as TS, WT, and GBB, relying solely on the establishment of conservation farms to protect diversity may yield limited results. Although creating conservation farms is a straightforward and widely used approach, it can sometimes lead to the loss of specific gene frequencies due to spatial constraints. Populations with low diversity can adopt various strategies to conserve genetic variation. In addition to establishing conservation farms, cryopreservation of semen or embryos can be employed to reduce the frequency of inbreeding [39]. With the revolution in biological technologies, conservation techniques such as DNA preservation, oocyte freezing, cloning, and the cryopreservation of embryonic stem cells have progressively advanced. Research focusing on the genetic mapping of genes associated with desirable traits, as well as gene isolation and cloning, has also increased. In the future, studies and practices related to conserving the genetic diversity of Tibetan sheep will need to be further developed [40].

## CONCLUSION

In all populations, instances of inbreeding have occurred, with some individuals in certain populations experiencing severe inbreeding. This has resulted in low genetic diversity within these groups. Therefore, targeted breeding management and strategies to conserve genetic diversity should be implemented.

## Figures and Tables

**Table 1:** Number information of SNP loci in Tibetan sheep populations

Populations	Number of SNPs
TS	879,801
KC	1,050,507
GJ	837,855
QK	900,856
OL	756,812
WT	992,538
ZSJ	760,518
GBW	920,429
GBB	765,414
AW	1,043,374
HB	1,099,861
Total	10,884,554

SNP, single nucleotide polymorphism; TS, Tao sheep; KC, Kecai sheep; GJ, Ganjia sheep; QK, Qiaoke sheep; OL, Oula sheep; WT, Tianjun white sheep; ZSJ, Zhashijia sheep; GBW, Gangba white sheep; GBB, Gangba black sheep; AW, Awang sheep; HB, Huoerba sheep.

**Table 2:** Number of ROHs in Tibetan sheep populations

Populations	Class A: 0.5–1 Mb	Class B: 1–3 Mb	Class C: >3 Mb	Mean	SD
TS	4,765 (92.72%)	374 (7.28%)	-	285	318.6
KC	1,992 (93.35%)	141 (6.61%)	1 (0.04%)	112.3	197.6
GJ	5,856 (86.14%)	925 (13.61%)	17 (0.25%)	424.9	494.2
QK	5,511 (90.97%)	545 (9.00%)	2 (0.03%)	336.6	345.2
OL	2,706 (90.41%)	287 (9.59%)	-	176.1	320.1
WT	6,200 (93.73%)	414 (6.26%)	1 (0.01%)	347.7	268.6
ZSJ	764 (97.32%)	21 (2.68%)	-	65.4	148.1
GBW	1,852 (96.16%)	74 (3.84%)	-	120.4	154.4
GBB	1,974 (86.31%)	311 (13.6%)	2 (0.09%)	175.9	382.7
AW	2,748 (94.76%)	152 (6.24%)	-	161.1	206.6
HB	1,588 (95.32%)	78 (4.68%)	-	92.6	106.2

Mean, the average number of ROHs per Tibetan sheep individual.

ROH, runs of homozygosity; SD, standard deviation; TS, Tao sheep; KC, Kecai sheep; GJ, Ganjia sheep; QK, Qiaoke sheep; OL, Oula sheep; WT, Tianjun white sheep; ZSJ, Zhashijia sheep; GBW, Gangba white sheep; GBB, Gangba black sheep; AW, Awang sheep; HB, Huoerba sheep.

**Table 3:** ROH length statistics of the Tibetan sheep population

Items	Mean (Mb)	SD	CV
TS	0.6861	0.2061	30.03935286
KC	0.6733	0.21	31.18966285
GJ	0.7518	0.3106	41.3141793
QK	0.7009	0.2327	32.8023682
OL	0.7094	0.2422	34.14152805
WT	0.6713	0.1945	28.97363325
ZSJ	0.63	0.154	24.44444444
GBW	0.6456	0.1563	24.21003717
GBB	0.7477	0.3111	41.60759663
AW	0.6639	0.1763	26.5552041
HB	0.6523	0.1658	25.41775257

Mean (Mb), the average length of ROH per Tibetan sheep individual.

ROH, runs of homozygosity; SD, standard deviation; CV, coefficient of variation; TS, Tao sheep; KC, Kecai sheep; GJ, Ganjia sheep; QK, Qiaoke sheep; OL, Oula sheep; WT, Tianjun white sheep; ZSJ, Zhashijia sheep; GBW, Gangba white sheep; GBB, Gangba black sheep; AW, Awang sheep; HB, Huoerba sheep.

**Table 4:** Statistical table of F_ROH_ in Tibetan sheep population

Items	FROH				

	Mean	SD	CV	Max	Min
TS	0.0990	0.0448	45.2075	0.1644	0.0001
KC	0.0154	0.0292	189.7671	0.1302	0.0001
GJ	0.0614	0.0750	122.0951	0.2168	0.0001
QK	0.0456	0.0504	110.5284	0.1869	0.0001
OL	0.0261	0.0510	195.8857	0.2090	0.0001
WT	0.0411	0.0351	85.5233	0.1487	0.0007
ZSJ	0.0091	0.0210	231.3192	0.0747	0.0001
GBW	0.0160	0.0210	130.7889	0.0612	0.0001
GBB	0.0280	0.0659	235.7643	0.2497	0.0002
AW	0.0214	0.0291	135.8001	0.1274	0.0001
HB	0.0128	0.0151	118.0060	0.0461	0.0001

F_ROH_, genomic inbreeding coefficient based on runs of homozygosity.

Mean (F_ROH_), the average length of F_ROH_ per Tibetan sheep individual.

ROH, runs of homozygosity; SD, standard deviation; CV, coefficient of variation; TS, Tao sheep; KC, Kecai sheep; GJ, Ganjia sheep; QK, Qiaoke sheep; OL, Oula sheep; WT, Tianjun white sheep; ZSJ, Zhashijia sheep; GBW, Gangba white sheep; GBB, Gangba black sheep; AW, Awang sheep; HB, Huoerba sheep.

**Table 5:** Diversity table for Tibetan sheep populations

Sample	Ho	He	F_IS
TS	0.181	0.297	0.392
KC	0.188	0.303	0.379
GJ	0.188	0.300	0.373
QK	0.191	0.291	0.344
OL	0.182	0.301	0.396
WT	0.199	0.306	0.350
ZSJ	0.173	0.298	0.418
GBW	0.176	0.298	0.408
GBB	0.174	0.295	0.409
AW	0.187	0.290	0.356
HB	0.183	0.300	0.389

F_IS, Fixation Index within subpopulations is also known as inbreeding coefficient within subpopulations.

Ho, observed heterozygosity; He, expected heterozygosity; TS, Tao sheep; KC, Kecai sheep; GJ, Ganjia sheep; QK, Qiaoke sheep; OL, Oula sheep; WT, Tianjun white sheep; ZSJ, Zhashijia sheep; GBW, Gangba white sheep; GBB, Gangba black sheep; AW, Awang sheep; HB, Huoerba sheep.

## Data Availability

Raw FASTQ files for whole-genome sequencing were deposited in the NCBI Sequence Read Archive (SRA) and have been assigned BioProject accession number: PRJNA1138910, and argali DNA illumina sequencing accession number: SRX15480971.

## SUPPLEMENTARY MATERIAL

**Supplement 1**. Sampling location information of Tibetan sheep.

**Supplement 2**. Tibetan sheep populations genome variation annotation results.
