# Supplementary material for: Detailed analysis of inbreeding in Tibetan sheep populations based on genome re-sequencing
Source: Anim Biosci. 2026 Apr 2;39(7):250600. doi: 10.5713/ab.250600 (PMC13353113; doi:10.5713/ab.250600)
Supplement: Supplementary file 1 [file ab-250600-Supplementary-1.pdf]

Supplement 1. Sampling location information of Tibetan sheep

Breeds	Acronymic	Count	Sampling site	Altitude(m)	Longitude and latitude
**Tao sheep**	TS	20	Liulin Town, Zhuoni County, Gannan Tibetan Autonomous Prefecture, Gansu Province	2887	N:34°64'97"<br>E:103°52'386"
**Kecai sheep**	KC	20	Zhaxi Bennang Cooperative Society in Kecai Town, Xiahe County, Gansu Province	3238	N:34°63'72"<br>E:102°22'319"
**Ganjia sheep**	GJ	20	Xike Village, Ganjia Town, Maqu County, Gannan Tibetan Autonomous Prefecture, Gansu Province	3022	N:35°32'49"<br>E:102°40'802"
**Qiaoke sheep**	QK	20	Galniang Village, Luqu County, Gannan Tibetan Autonomous Prefecture, Gansu Province	3498	N:35°42'11"<br>E:102°42'210"
**Oula sheep**	OL	20	Darqing Village, Maqu County, Gannan Tibetan Autonomous Prefecture, Gansu Province	3501	N:33°51'31"<br>E:101°52'424"
**Tianjun white sheep**	WT	20	Xinyuan Town, Tianjun County, Haixi Mongolian and Tibetan Autonomous Prefecture, Qinghai Province	3331	N:37°28'46"<br>E:99°10'188"
**Zhashijia sheep**	ZSJ	20	Yuegai Town, Qumalai County, Yushu Tibetan Autonomous Prefecture, Qinghai Province	4269	N:34°14'87"<br>E:95°80'422"
**Gangba white sheep**	GBW	20	Dongga, Kongma Township, Gangba County, Shigatse City, Xizang Autonomous Region	4401	N:28°45'89"<br>E:88°61'37"
**Gangba black sheep**	GBB	20	Gangba Town, Gangba County, Shigatse City, Xizang Autonomous Region	4555	N:28°24'66"<br>E:88°41'36"
**Awang sheep**	AW	20	Awang Township, Gongjue County, Changdu, Xizang Autonomous Region	4643	N:30°12'10"<br>E:98°63'098"
**Huoerba sheep**	HB	20	Rima Village, Zhongba County, Shigatse City, Xizang Autonomous Region	4614	N:33°51'31"<br>E:101°52'424"
