# Supplementary material for: Detailed analysis of inbreeding in Tibetan sheep populations based on genome re-sequencing
Source: Anim Biosci. 2026 Apr 2;39(7):250600. doi: 10.5713/ab.250600 (PMC13353113; doi:10.5713/ab.250600)
Supplement: Supplementary file 2 [file ab-250600-Supplementary-2.pdf]

Supplement 2. Tibetan sheep populations genome variation annotation results

Sample	downstream	exonic	exonic; splicing	intergenic	intronic	splicing	upstream	upstream; downstream	UTR3	UTR5	UTR5; UTR3
**TS**	3480.1 (0.6917%)	1791.4 (0.356%)	2.35 (0.0005%)	349669.7 (69.4973%)	171351.25 (34.0563%)	74.35 (0.0148%)	3301.55 (0.6562%)	92.25 (0.0183%)	1391.4 (0.2765%)	347.1 (0.069%)	17.1 (0.0034%)
**KC**	3307.6 (0.6574%)	1798.1 (0.3574%)	2.25 (0.0004%)	314740.65 (62.5551%)	159668.85 (31.7344%)	71.85 (0.0143%)	3108.3 (0.6178%)	93.4 (0.0186%)	1326.7 (0.2637%)	337.05 (0.067%)	16.3 (0.0032%)
**GJ**	3481.75 (0.692%)	1767.85 (0.3514%)	2.1 (0.0004%)	355029.5 (70.5626%)	173731.2 (34.5293%)	75.25 (0.015%)	3317.25 (0.6593%)	92.8 (0.0184%)	1422.1 (0.2826%)	350.55 (0.0697%)	17.15 (0.0034%)
**QK**	3489.55 (0.6936%)	1846.1 (0.3669%)	2.7 (0.0005%)	342089.1 (67.9907%)	170705.55 (33.928%)	73.2 (0.0145%)	3292.25 (0.6543%)	98.85 (0.0196%)	1405.55 (0.2794%)	349.95 (0.0696%)	15.7 (0.0031%)
**OL**	3235 (0.643%)	1739.55 (0.3457%)	2.05 (0.0004%)	316581.2 (62.9209%)	157491.25 (31.3016%)	68.85 (0.0137%)	3077.1 (0.6116%)	92.75 (0.0184%)	1300.6 (0.2585%)	326.95 (0.065%)	15.45 (0.0031%)
**WT**	3631.2 (0.7217%)	1912.45 (0.3801%)	2.3 (0.0005%)	358732.85 (71.2986%)	178671.9 (35.5113%)	76.2 (0.0151%)	3444.65 (0.6846%)	100.45 (0.02%)	1478.55 (0.2939%)	364.85 (0.0725%)	19 (0.0038%)
**ZSJ**	3083.8 (0.6129%)	1637.25 (0.3254%)	2 (0.0004%)	306092.35 (60.8363%)	151469 (30.1047%)	65.45 (0.013%)	2920.5 (0.5805%)	85.05 (0.0169%)	1235.4 (0.2455%)	322.05 (0.064%)	16.8 (0.0033%)
**GBW**	3277.25 (0.6514%)	1716.6 (0.3412%)	2.4 (0.0005%)	322187.25 (64.0351%)	161495.95 (32.0975%)	70.4 (0.014%)	3143.9 (0.6249%)	89.75 (0.0178%)	1346.2 (0.2676%)	336.8 (0.0669%)	16.45 (0.0033%)
**GBB**	3140.3 (0.6241%)	1681.1 (0.3341%)	2.9 (0.0006%)	309862.8 (61.5856%)	153764.3 (30.5609%)	67.9 (0.0135%)	3004.4 (0.5971%)	86.75 (0.0172%)	1270.35 (0.2525%)	324 (0.0644%)	16.25 (0.0032%)
**AW**	3399.65 (0.6757%)	1843.2 (0.3663%)	2.8 (0.0006%)	328075.35 (65.2054%)	164666.2 (32.7276%)	74.15 (0.0147%)	3245.65 (0.6451%)	95.45 (0.019%)	1370.65 (0.2724%)	349.7 (0.0695%)	18.5 (0.0037%)
**HB**	3232.3 (0.6424%)	1797.4 (0.3572%)	2.45 (0.0005%)	306787.5 (60.9744%)	155381.1 (30.8822%)	72.3 (0.0144%)	3124.7 (0.621%)	91.55 (0.0182%)	1298.85 (0.2581%)	339.6 (0.0675%)	15.9 (0.0032%)
